# Natural Product Chemistry and Biological Research

**DOI:** 10.3390/ijms25073774

**Published:** 2024-03-28

**Authors:** Francisco Les, Marta Sofía Valero, María Pilar Arruebo

**Affiliations:** 1 Department of Pharmacy, Faculty of Health Sciences, Universidad San Jorge, 50830 Zaragoza, Spain; 2 Instituto Agroalimentario de Aragón-IA2, CITA-Universidad de Zaragoza, 50059 Zaragoza, Spain; msvalero@unizar.es (M.S.V.); parruebo@unizar.es (M.P.A.); 3 Departamento de Farmacología, Fisiología y Medicina Legal y Forense, Universidad de Zaragoza, 50009 Zaragoza, Spain

Natural products are substances found in nature that have not been significantly modified by humans. They can originate from plants, animals, fungi, and microorganisms. The chemical composition of natural products is highly diverse and can include a wide range of compounds. Natural products have gained popularity in various industries, including pharmaceuticals, cosmetics, and agri-food, due to the growing demand for more sustainable and healthier options [1,2].

Among these, plants stand out as an endless source of active principles and the basis of pharmacotherapy. These active principles can be extracted from various parts of plants, such as leaves, flowers, roots, seeds, or fruits. Some notable molecules include morphine, an analgesic found in opium from the poppy plant (*Papaver somniferum*); artemisinin, an antimalarial from the plant *Artemisia annua*; and caffeine, a stimulant present in coffee beans, tea leaves, and other plants. Additionally, plants contain essential oils such as limonene from citrus fruits, linalool from lavender or laurel, or menthol from mint, used in perfumery, aromatherapy, or pharmaceuticals [3,4].

Among animal-derived products, some stand out, such as keratin, present in hair, nails, and animal horns, used in hair and nail care products. Uric acid, found in animal viscera, is used in the manufacture of many cosmetic products. Although animal venoms can be dangerous, they may contain compounds with medicinal properties. Some snake venoms contain proteins that have been studied to develop drugs to treat diseases such as chronic pain, and captopril, an antihypertensive drug, was obtained from the venom of the *Bothrops jararaca* snake [5].

Marine-derived products also stand out. From algae, notable compounds include carrageenans from *Chondrus crispus*, agar-agar from *Gelidium*, with important applications in industries as thickeners and gelling agents, and Spirulina, an important source of proteins, vitamins, and minerals, known for its high nutritional value. Marine organisms, such as sponges, corals, and marine microorganisms, produce compounds with pharmacological properties. For example, some cancer drugs and antibiotics come from marine organisms. Other marine-derived compounds include Omega-3, found in fatty fish like salmon, known for its cardiovascular and brain health benefits, and collagen, found in the skin and scales of fish and other marine animals, used in skincare products [6,7].

Compounds from fungi include penicillin, an antibiotic from the *Penicillium* fungus, and lovastatin from *Aspergillus terreus*, used in the production of cholesterol-lowering medications [8].

Ongoing research in the field of natural products aims to identify new compounds and to better understand their mechanisms of action. These compounds can be used in drug formulations, nutritional supplements, and personal care products. However, it is important to note that not all natural products are safe or effective, and rigorous scientific research is necessary to evaluate their benefits and risks.

It is advisable to consult a healthcare professional before incorporating new supplements or natural products into your routine, especially if you are taking medications or have pre-existing health conditions.

These are just a few examples, as the diversity of natural products and their chemical components is vast. It is important to note that although these compounds are found in nature, some can be synthesized or modified in the laboratory to produce more purified or enhanced versions.

As the editor of this Special Issue, I want to express my gratitude to the researchers, scientists, and academics who have contributed their innovative work. Their dedication to scientific excellence has enriched this Special Issue, providing a platform for knowledge exchange and promoting interdisciplinary collaboration.

I hope this Special Issue serves as a valuable resource for the scientific community, inspiring new research and encouraging the application of acquired knowledge in addressing global challenges in health and the environment.
